# Book Reviews

**Published:** 1915-03

**Authors:** 


### BOOK REVIEWS

A TEXT-BOOK OF DISEASES OF THE NOSE AND THROAT. By D. Braden Kyle, A. M., M. D., Professor of Laryngology and Rhinology, Jefferson Medical College, Philadelphia. Fifth edition, thoroughly revised and enlarged. Octavo of 856 pages with 272 illustrations, 27 of them in colors. Philadelphia and London: W. B. Saunders Company, 1914. Cloth, $4.50 net.

This is practically a new book because of the extensive and careful revisions that have been made. Vaccine and Bacterio Therapy have been described in their latest manifestations, while Syphilis and Accessory Sinus diseases are presented devoid of speculation and with precision.

The author has already led the way in the consideration of nervous reflexes in relation to the nose and in this edition the chapter is clear and illuminating. The subject of Voice and Speech is well handled and the place of acoustics in the study of defects in speech and diseases of the voice organs is indicated.

Even the operations on the tonsils and nasal septum which we sometimes think have had every possible thing said about them, are cleverly discussed and simplifying suggestions made.

As to the instrumentation of examination not so much can be said. Perhaps the author's experience in recommending apparatus of this sort has run against a snag of personal equation so strongly that he hesitates to meet that obstacle again in a positive way. There seems room for simplification of the instruments and surely there is opportunity for elimination of some clumsy things. This is really a personal matter with each student and operator, however, and in no wise detracts from the value of the book.

It is good to have a unified work upon the nose and throat and to feel confident that the author speaks conscientiously as one having authority. R. R. D.

